# Psychiatrists and other medical professionals in Belgium show a substantial lack of knowledge about poverty

**DOI:** 10.1192/j.eurpsy.2023.2430

**Published:** 2023-07-17

**Authors:** Kirsten Catthoor, Marc De Hert, Hans De Loof, Ingrid Jongeneelen, Yves Wuyts, Kris Van den Broeck

**Affiliations:** 1 Estates-General of Mental Health, Kortenberg, Belgium; 2 Flemish Association of Psychiatry, Kortenberg, Belgium; 3 Collaborative Antwerp Psychiatric Research Institute (CAPRI), University of Antwerp, Antwerp, Belgium; 4 University Psychiatric Centre, Katholieke Universiteit Leuven, Kortenberg, Belgium; 5 Department of Neurosciences, Centre for Clinical Psychiatry, Katholieke Universiteit Leuven, Leuven, Belgium; 6 Leuven Brain Institute, Katholieke Universiteit Leuven, Leuven, Belgium; 7 Antwerp Health Law and Ethics Chair, University of Antwerp, Antwerp, Belgium; 8 Department of Pharmaceutical Sciences, University of Antwerp, Antwerp, Belgium; 9 UilenSpiegel, Brussels, Belgium; 10 Zorgnet-Icuro, Brussels, Belgium; 11 Ziekenhuis Netwerk Antwerpen, Psychiatrisch Ziekenhuis Stuivenberg, Antwerp, Belgium

**Keywords:** health professionals, psychiatrists, general practitioner, mental health, poverty, 10-point action plan, knowledge

## Abstract

The bidirectional relationship between poverty and poor physical and mental health is well-known. All physicians should have sufficient knowledge on poverty as a social determinant and its impact on (mental) health. The knowledge of poverty in physicians is seldom investigated. An online and paper survey was circulated in March/April 2022 in Belgium, to assess physician’s opinions about and attitudes toward patients in poverty. Not only was interest in the subject rather low, but there were also substantial contradictions in the responses. The lack of knowledge about poverty among physicians leads to reduced quality of medical care for this target group. This is an individual medical–ethical and societal problem. We suggest 10 point-action plan for policymakers, educational institutions, and physicians.

The bidirectional relationship between poverty (the lack of financial resources to provide life necessities) and poor physical and mental health has been extensively described [1]. Both poverty and global health are complex in nature, multidimensional, and intertwining. Poverty can induce poor mental health through different pathways. Worries and uncertainty about living conditions lead to higher psychosocial stress. Detrimental environmental factors (pollution, temperature extremes, and night noise), disturbed sleep, chronic pain, malnutrition, and traumatic early life events compromise cognitive development and mental functioning. In the opposite direction is endangered mental health responsible for the development of impoverished thinking and wrong economic decisions, leading to financial decline and dependency on social security incomes [2].

Poverty is not only a shortfall of money, but also a scarcity of psychological possibilities, and diminished educational and job opportunities. Cognitive resources are under pressure due to attentional capture, intrusive thoughts, and impaired insight [3]. Children growing up in poverty have poorer outcomes in terms of education, behavior, and health, and those consequences remain visible into adulthood [4].

The United Nations Sustainable Development Goals 2022 report states that the COVID-19 pandemic wiped out more than 4 years of progress on poverty eradication and pushed 75–95 million more people into extreme poverty in 2022 [5]. Financial problems will probably continue to increase in all population groups, due to rising energy costs, unstable political situations, and climate change. The mental health of people in energy poverty (not being able to warm your house) declines significantly [6], with odds for depression and anxiety rising by 49%. There are no arguments that increased economic growth could reduce financial inequality for people with mental vulnerability. Spivak [7] shows that patients with serious mental illness (SMI) who struggle daily with financial hardship exhibit more psychiatric symptoms. The consequences of both psychiatric and financial problems remain persistent over long stretches of time.

Therefore, it should be of paramount importance that all physicians, general practitioners (GPs), and psychiatrists in particular, have sufficient knowledge on poverty as a social determinant, its impact on (mental) health, the consequences for treatment and noncompliance, and how to care for this population. Remarkably, very little attention is paid to the subject in the scientific literature. The knowledge of poverty in physicians is seldom investigated. Some papers report on focus groups to better understand working with poverty and mental illness [8], while others on learning activities, like poverty simulations, to enhance clinicians’ awareness and empathy [9]. There are no studies assessing the knowledge of poverty in medical practitioners.

Advocacy groups from patients with lived experience in Belgium have sounded the alarm on the importance of poverty and its impact on mental health treatment in the task force group “Poor makes sick, sick makes poor” from the Estates-General of Mental Health (EGMH). The EGMH is an organization of all interested stakeholders within the extended mental health sector. It aspires to arrive at a shared vision of the current strengths and vulnerabilities within the mental health sector and translate them into policy recommendations and priorities for change.

Persons with lived experience in the “Poor makes sick, sick makes poor” working group indicated that doctors lack sufficient knowledge about poverty in daily life. Physicians are not aware of the out of pocket costs of medication, psychological, and other essential nonmedical interventions and do not make sufficient referrals to social services. The working group decided to initiate a survey on the topic. In collaboration with De Artsenkrant/Le Journal du Médecin – a weekly Belgian journal for medical practitioners with a circulation of 25,000 copies – an online and paper survey was circulated in March/April 2022 to assess physician’s opinions about and attitudes toward patients in poverty.

Remarkably, the interest in the study was low: only 395 valid responses were collected. Findings suggest that nonresponse is not arbitrary. The majority of respondents were GPs (*n* = 209; 53%), psychiatrists (*n* = 34, 9%), and older than 60 (*n* = 162, 41%). Compared to younger doctors, the latter reported having more often experienced poverty in their own lives. GPs and psychiatrists are generally more involved with the familial, social, and professional life of their patients, and poverty may be more visible. The characteristics of the sample, therefore, suggest some level of expertise in our respondents.

Further data analysis revealed that most respondents acknowledge the intergenerational nature of poverty (71%), its situational origin (72%), and the barriers to equally qualitative (59%), timely (66%), and affordable care (80%). According to one in three, people in poverty have their priorities wrong, and 14% of the respondents claim that people in poverty make insufficient efforts to change their situation. Nevertheless, respondents acknowledge that adequate care for people in poverty is hindered by administrative burdens (77%) and lack of time by healthcare professionals (61%).

Only 10 and 28% always or often initiate a discussion with the patient regarding financial problems, and 17% do this seldom or never. Despite this, respondents stated that they often (41%) or always (41%) take the patient’s financial status into account when prescribing medication. According to the participants, patients in poverty often (16%) or always (7%) end up with cheaper but suboptimal treatment. Luckily, patients in poverty remain welcome at most practitioners (90%). Some physicians often (24%) or always (15%) offer them a consultation without fee overrun. GPs and respondents with personal experience with poverty were more willing to adjust their treatment proposal and payment options.

Though half of the respondents (53%) admitted that they find it (very) difficult to work with patients in poverty, 72% were convinced that they have sufficient knowledge to work with them. It is, however, unclear where they have acquired this knowledge: 78% indicated that poverty was not included in their medical training; 50% indicated that they would participate in a training on the subject if that was available. When asked to rank five commonly given medications by personal cost to the patient (after reimbursement), only 12% gave the correct answer. This is a compelling finding, given the impact poverty has – both as a vulnerability and as a sustaining factor – for illness in general and psychopathology in particular.

The lack of knowledge about poverty among doctors leads to reduced quality of medical care for this target group. This is an individual medical–ethical and societal problem, due to increased total costs. It is therefore essential to formulate an appropriate approach to change. We suggest 10 point-action plan for policymakers, educational institutions, and physicians:

1. As a government, realize health in all policies such as housing first, sufficient green space in every residential area, opportunity for free sports and exercise, and so on, as indispensable tools in improving the health of people in poverty.
2. As a government, encourage and invest in integrated care, so that patients in poverty will be spontaneously referred to social services. Children, adolescents, and their parents should be prioritized. Conversely, the right to affordable care should be without administrative burden safeguarded for these patients.
3. Include education on poverty, and its effects on patients’ well-being and health, in any basic training for physicians and all other health professionals.
4. Give physicians frequent updates on the cost of treatment: the price of technical examinations (radiology and laboratory tests), the price of medication, and nonpharmacological interventions (physiotherapy, psychotherapy, dietary advice, and smoking cessation counseling). Encourage physicians to actively search for treatment costs in dealing with patients in poverty.
5. Instruct physicians on how patients in poverty can obtain quality healthcare at par with the general population by taking financial constraints into account.
6. Raise physician’s awareness to advocate for better reimbursement or free treatment for patients in poverty, toward the government and other agencies.
7. Encourage doctors and hospitals or other medical institution to accept staggered payments from patients in a precarious financial situation.
8. Set up peer-to-peer groups and in-house therapeutic support for physicians heavily involved in caring for patients in poverty. They are a high-risk group for developing moral injury.
9. Develop an easily manageable screening tool for the detection of patients at risk and make it freely available to health workers.
10. Include buddy work and experts by experience in all forms and levels of medical care, as they are important supports for patients in poverty and doctors.

Article 25 of the Universal Declaration of Human Rights is very clear on poverty and health: “Everyone has the right to a standard of living adequate for the health and well-being of himself and of his family, including food, clothing, housing and medical care and necessary social services, and the right to security in the event of unemployment, sickness, disability, widowhood, old age or other lack of livelihood in circumstances beyond his control” [10]. There is no doubt that this article is being violated many times, every day, not only in underdeveloped, but also in most developed countries in the world. Physicians have a moral duty to denounce and combat this social injustice and do efforts to provide every patient in poverty standard quality health care on a par with the general population in their country.

## References

[1] Ridley M, Rao G, Schilbach F, Patel V. Poverty, depression, and anxiety: causal evidence and mechanisms. Science. 2020;370(6522):eaay0214. doi:10.1126/science.aay0214. 33303583

[2] Lund C, Brooke-Sumner C, Baingana F, Baron EC, Breuer E, Chandra P, et al. Social determinants of mental disorders and the sustainable development goals: a systematic review of reviews. Lancet Psychiatry. 2018;5(4):357–69. doi:10.1016/S2215-0366(18)30060-9. 29580610

[3] Mani A, Mullainathan S, Shafir E, Zhao J. Poverty impedes cognitive function. Science. 2013;341(6149):976–80. doi:10.1126/science.1238041. 23990553

[4] Duncan GJ, Magnuson K, Votruba-Drzal E. Moving beyond correlations in assessing the consequences of poverty. Annu Rev Psychol. 2017;68:413–34. doi:10.1146/annurev-psych-010416-044224. 27648987 PMC6108837

[5] The Sustainable Development Goals Report 2022, United Nations.p/3. https://unstats.un.org/sdgs/report/2022/The-Sustainable-Development-Goals-Report-2022.pdf.

[6] Bentley R, Daniel L, Li Y, Baker E, Li A. The effect of energy poverty on mental health, cardiovascular disease and respiratory health: a longitudinal analysis. Lancet Reg Health West Pac. 2023;35:100734. doi:10.1016/j.lanwpc.2023.100734. 37424688 PMC10326697

[7] Spivak S, Cullen B, Eaton WW, Rodriguez K, Mojtabai R. Financial hardship among individuals with serious mental illness. Psychiatry Res. 2019;282:112632. doi:10.1016/j.psychres.2019.112632. 31690462

[8] Thomas F, Hansford L, Wyatt K, Byng R, Coombes K, Finch J, et al. An engaged approach to exploring issues around poverty and mental health: a reflective evaluation of the research process from researchers and community partners involved in the DeStress study. Health Expect. 2021;24:113–21. doi:10.1111/hex.13065. 32449304 PMC8137483

[9] Murray PM, Sepulveda A, Baird J. Longitudinal impact of a poverty simulation on healthcare practitioners’ attitudes towards poverty. J Pediatr Nurs. 2022;64:24–30. doi:10.1016/j.pedn.2022.01.016. 35131716

[10] Universal Declaration of Human Rights, United Nations, Article 25: https://www.un.org/en/about-us/universal-declaration-of-human-rights.

